# Fabrication of Antimicrobial Cellulose and Silver Niobate Aerogels for Enhanced Tissue Regeneration

**DOI:** 10.1021/acsomega.5c00351

**Published:** 2025-04-11

**Authors:** Marcela Piassi Bernardo, Mauricio Foschini, Ana Carolina Costa Santos, Carlos Ueira Vieira, Natieli Saito, Maria Eduarda Costa Mundim, Osmando Ferreira Lopes, Daniel Pasquini

**Affiliations:** † Institute of Chemistry, Federal University of Uberlândia, Av João Naves de Ávila, Uberlândia, MG CEP 38400-902, Brazil; ‡ Physics Institute, Federal University of Uberlandia, Av João Naves de Ávila, Uberlândia, MG CEP 38400-902, Brazil; § Genetics Laboratory, Institute of Biotechnology, Federal University of Uberlandia, Rua Ceará, Uberlândia, MG CEP: 38402-018, Brazil; ∥ Nanobiotechnology Prof. Dr. Luiz Ricardo Goulart Filho Laboratory, Institute of Biotechnology, Federal University of Uberlandia, Rua Ceará, Uberlândia, MG CEP: 38402-018, Brazil

## Abstract

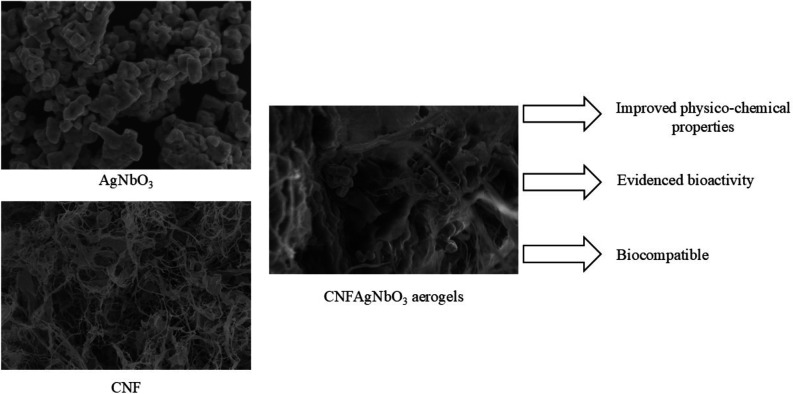

Aging, trauma, infection, illness, and accidents can lead to the disruption of various human tissues, including skin, bone, and cartilage. Tissue engineering aims to promote the growth of cells and tissues within the human body, with scaffolds serving as vehicles to deliver a combination of mechanical and molecular signals to create new tissues for body reconstruction. Composite materials have gained significant attention as an attractive alternative for scaffolding due to their ability to enhance multiple material properties. For instance, cellulose nanofibers are known for their high specific surface area, flexibility, and elasticity. However, their limited bioactivity and slow degradation rates restrict their suitability for tissue engineering applications. In contrast, niobium-based materials, which are biocompatible and nontoxic, have been underexplored in this field. In this study, silver niobate is investigated for the first time as a component of a composite material designed to provide biological activity to an aerogel, thereby creating a multifunctional scaffold for tissue regeneration. Silver niobate nanoparticles were successfully synthesized and characterized by X-ray diffraction (XRD), Fourier transform infrared (FTIR), and scanning electron microscopy (SEM). The composite aerogels demonstrated improved thermal stability, hydrophilicity, bioactivity, and antimicrobial activity against *Staphylococcus aureus*. Additionally, the developed aerogels showed no cytotoxic effects on primary dermal fibroblast (HDFn) cells. These findings suggest that the silver niobate-based aerogel composite holds significant potential for applications in tissue regeneration, offering a promising avenue for the development of advanced biomaterials in regenerative medicine.

## Introduction

Aging, trauma, infection, illness, and accidents may cause disruption of different human tissues, like skin, bone, and cartilage.^1^ Usually, when the human tissues have their integrity compromised, the body initiates a complex reaction to repair the damaged tissue and restore its function.^2^ However, large damage to human tissues cannot be repaired by itself, requiring the use of scaffolds or dressings.^3^

Tissue engineering aims to promote the growth of cells and tissue in human body and scaffolds are the vehicle to deliver a combination of mechanical and molecular signals to create a new tissue for the human body reconstruction.^4^ The scaffolds mimic the extracellular matrix in natural tissues, and they are used as a matrix for cell growth, proliferation, and differentiation, creating an ideal environment for the growth of tissues.^5^ The scaffolds need to exhibit essential characteristics such as biocompatibility or little immune rejection and suitable physicochemical stability after body implantation; degradation rate compatible with the tissue growth rate; and textural and mechanical properties similar to the target tissue.^6^

Biomaterials play a key role in regenerative medicine, with metals, ceramics, and polymers offering distinct advantages for tissue restoration.^7^ However, the complex requirements of tissue regeneration are best met by composite materials, which combine the strengths of these classes to achieve optimal performance.^6^ Composite materials are an attractive alternative due to their unique and improved properties compared to neat materials, such as ceramic, metal, and polymer.^8^ In this sense, composite biomaterials may result in a scaffold with improved degradation rate, mechanical properties, and bioactivity.^9^

Niobium (Nb)-based compounds are rarely investigated outside the catalysis field.^10^ However, these materials are drawing attention for biomaterial applications due to their intrinsic properties.^11^ Silver niobate, for example, possesses biocompatibility, high chemical stability, and suitable mechanical properties, making it a promising candidate for biomaterial applications.^12^ The antibacterial nature of silver ions released from silver niobate prevents infection, a critical aspect in regenerative medicine.^13^ In addition, Nb can increase cell adhesion and proliferation during the regeneration process.^14^

To develop composites, several types of polymeric materials are available to support tissue regeneration. Usually, natural polymers (like alginate and chitosan) fail in providing adequate mechanical strength or do not contain cell-binding moieties that promote cell attachment and infiltration.^15^ On the other hand, synthetic polymers (like poly(lactic acid) (PLA), polycaprolactone (PCL), and poly(vinyl alcohol) (PVA)) have strong mechanical properties; however, the biocompatibility and cell attachment are poor for tissue engineering.^16^ In this scenario, cellulose arises as an excellent alternative for tissue engineering, since this polymer overcomes several drawbacks of natural polymers.^17^

Cellulose is a polysaccharide composed of a linear chain of d-glucose units linked by β-(1,4) glycosidic bonds.^18^ It is considered as the most abundant polysaccharide on earth; therefore, the application of this material has a great economical advantage.^19^ Besides that, cellulose is biocompatible, nonimmunogenic, nontoxic, and exhibits an interconnected porous structure, which are remarkable features for its applications in tissue engineering.^20^ Specifically, cellulose nanofiber (CNF) has been used for tissue engineering due to its high specific surface area and abundant polar groups.^21^ CNF is obtained from chemical removal of hemicellulose followed by acid hydrolysis, resulting in a material with crystalline and amorphous regions. The crystalline region contributes to the stiffness and elasticity, while the amorphous region contributes to the flexibility and plasticity of the materials.^9^ However, the mechanical properties, water uptake capacity, degradation rates, and low bioactivity of cellulose may not be adequate for tissue engineering.^22^ The development of scaffolds based on cellulose and silver niobate may mitigate the negative aspects of the polymer and provide adequate bioactivity to the desired scaffold.^1^

Scaffolds for tissue engineering should be designed using pores with adequate size to support three-dimensional (3D) cell–cell contact and allow good diffusion of nutrients, oxygen, and bioactive factors for cell survival and growth.^8^ Aerogels hold attractive properties for application in tissue engineering, like interconnected high porosity, high specific surface area, and low density,^9,19^ creating an adequate environment for cell attachment and proliferation.^23,24^

Different methods are available to fabricate aerogel scaffolds, like freeze-drying, phase separation, particulate leaching, and gas foaming.^25^ In the field of medical applications, aerogels have been studied for drug delivery, wound-healing dressings, biosensors for diagnostics, and as scaffold for regenerative medicine.^26^ Aerogels can be designed to have different shapes to suit the properties of the desired tissue to be regenerated without structural collapse or shrinkage.^27^ The singular biological properties and the possibility to customize the scaffold architecture and composition have attracted scientific attention to develop novel solutions for different tissue defects.^28^

Kamel et al.^29^ produced an aerogel based on cellulose/glucosamine loaded with rosuvastatin and reinforced Sr_3_B_2_O_6_, a bioactive ceramic. The authors noticed a mechanical increase of the scaffolds in comparison to neat cellulose aerogels. Besides that, the increase of the ceramic content delayed the release of rosuvastatin. On the other hand, the highest ability to increase MG-63 cells’ proliferation was achieved when a lower concentration of Sr_3_B_2_O_6_ was used. Chen et al.^30^ also used cellulose as polymer matrix for the development of aerogels blended with silk fibroin. The prepared aerogels had a microstructure ideal for cancellous bone repair and accelerated proliferation of human embryonic kidney cells. Osorio et al.^31^ used chemically cross-linked cellulose nanocrystals to produce aerogels that demonstrated growth of hydroxyapatite and led to increase in bone volume fraction, showing that cellulose can facilitate bone growth. Other polymers like poly(vinyl alcohol) (PVA),^32^ gelatin,^33^ and collagen/alginate^34^ have been used in aerogels’ composition for engineering of different tissues like cardiac, nerve, and bone.

Herein, we report the development and characterization of innovative composite materials by combining cellulose with silver niobate for tissue engineering applications. This specific combination is novel and has not been previously discussed in the literature.

This work addresses a significant gap in the literature by exploring the use of niobates (specifically silver niobate) in polymer composites. Traditionally, niobium has been utilized as a coating for metallic structures, making its application in scaffolds for tissue engineering a novel approach.

Different tissues can be beneficiated by the development of this kind of composite aerogels due to the great capacity of the aerogels to acquire different shapes, that can be suitable for different traumatized tissues. It was hypothesized that silver niobate can contribute to the enhancement of the physical properties of cellulose aerogels and provide an antimicrobial performance to the developed scaffolds. Thus, this study was aimed at establishing the relations between the structure, surface wettability, and cytotoxicity of cellulose/silver niobate aerogels, produced by freeze-drying. The presence of silver niobate in the aerogels was determined as the reason for the antibacterial efficiency against *Staphylococcus aureus*. The cytotoxicity of aerogels was assessed by Resazurin assay using human fibroblast cells.

## Materials and Methods

### Materials and Chemicals

Cellulose nanofiber (CNF) was provided by Suzano S/A (SP, Brazil); niobium ammonium oxalate was donated by Companhia Brasileira de Metalurgia e Mineração (CBMM) (MG, Brazil); and silver nitrate was purchased from Synth (SP, Brazil). All reagents were used as received. Deionized water (ρ > 18.2 MΩ cm) was obtained with a Barnstead Nanopure Diamond purification system (Thermo Fisher Scientific Inc., USA).

### Niobium Oxide (Nb_2_O_5_) Synthesis

5 mmol niobium ammonium oxalate was added to 100 mL of distillated water. To that suspension was added 6.7 mL of hydrogen peroxide (H_2_O_2_) at the molar ratio of 1:10 of Nb/H_2_O_2_, according to the oxidant peroxide method.^35,36^ The addition of H_2_O_2_ to niobium solution generated the formation of the niobium peroxocomplex, which was confirmed by the formation of a yellow solution. This solution was added to a Teflon jar and hydrothermally treated at 120 °C for 18 h. After the reaction, the material was washed twice with distillated water and once with isopropanol. The obtained material was dried at 50 °C for 5 h. The sample obtained from the niobium ammonium oxalate precursor was named Nb_2_O_5_.

### Silver Niobate (AgNbO_3_) Synthesis

AgNbO_3_ was synthesized following the methodology of Lu et al.^37^ Briefly, AgNbO_3_ powder was synthesized by the solid-state reaction method. 2.1 mmol AgNO_3_ and 1.0 mmol Nb_2_O_5_ were mixed by manual grinding (agate mortar and pestle) for 15 min. The mixture was calcined for 5 h in an alumina crucible at 880 °C. The sample was naturally cooled down to ambient temperature. The impurities were removed by successive treatment with concentrated HNO_3_ and H_2_O. The final powder was dried for 12 h in an oven at 60 °C.

### Aerogel Preparation

The CNF solution had 3% (w/w) of cellulose. For the aerogel preparation, silver niobate was dissolved in CNF solution to obtain composites with 10, 30, and 50% (w/w) of silver niobate related to cellulose mass present in the solution. The final solutions were mechanically stirred for 15 min and deposited in round pans, and then immediately frozen in liquid nitrogen and freeze-dried in LIOTOP-L101 at −70 °C and 0.01 mbar for 48 h. After freeze-drying, the aerogel sponges were removed from the pans and stored in a desiccator with silica gel. The aerogels were labeled as CNF_10_AgNbO_3_, CNF_30_AgNbO_3_, and CNF_50_AgNbO_3_.

### Materials Characterization

X-ray diffraction (XRD) measurements were performed using a Shimadzu XRD 6000 diffractometer, with Ni-filtered Cu Kα radiation (λ = 1.5405 Å), in the 2θ range from 10 to 70°, at a scan speed of 2° min^–1^. Fourier transform infrared (FTIR) spectroscopy analyses used a Bruker spectrometer in attenuated total reflectance (ATR) mode with a spectral resolution of 2 cm^–1^. Scanning electron microscopy (SEM) employed a JEOL microscope operating at 15 kV. The hydrophilic/hydrophobic surface characteristics of the films were investigated by contact angle measurements, using a contact angle meter (KSV Instruments, Helsinki, Finland). Images of water drops with an estimated volume of 5 μL were acquired and analyzed using a CCD video camera. The initial (*t* = 0 s) and equilibrium (*t* = 25 s) contact angles were calculated by averaging 5 individual measurements (*n* = 5) from 5 different film pieces. Thermogravimetric analysis (TGA) was performed with a Q400 analyzer (TA Instruments). Samples were dried for 24 h before the TGA tests. The experiments were conducted using an aluminum crucible in the temperature range from 25 to 600 °C (heating ramp of 10 °C min^–1^) using oxidizing atmosphere (60 mL min^–1^).

### Aerogel Characterization

The composite cellulose-based aerogels were incubated in simulated body fluid (SBF) to determine the material’s potential to induce the mineralization and therefore evaluate the bioactivity of the materials. The bioactivity assays were performed following the methodology proposed by Kokubo et al.^38^ The composite aerogels were cut into 1 × 1 × 0.3 cm^3^ samples, placed into plastic conical tubes, immersed in 35 mL of SBF (pH 7.4), and placed in an incubator at 36.5 °C for 4 weeks. After this time, the materials were removed from the solution, washed with distillated water, and dried in a desiccator without heating. The dry samples were characterized by SEM analysis combined with energy-dispersive spectrometry (EDS). All of the tests were performed in triplicate.

To determine the water uptake ability of the developed aerogels, the materials were weighed and recorded as *W*_0_ and then immersed in Milli-Q water to reach a swelling equilibrium at 37 °C. Then, the materials were removed from the water and the moisture on the surface of the material was quickly absorbed by the filter paper; the weight was recorded as *W*_t_. The water uptake ability was calculated as (*W*_t_ – *W*_0_)/*W*_t_ × 100%. All of the tests were performed in triplicate.

### In Vitro Antimicrobial Assays

The antimicrobial activities of the composite aerogels were tested against *S. aureus* (MECA) and *Escherichia coli* using the disk diffusion test method. Stock cultures of these microorganisms were kept in Falcon tubes at 36 °C during 24 h for cell growth. The resulting inoculum suspensions were diluted in 0.9% (w/v) NaCl solution until reaching 0.5 McFarland standard turbidity (10^8^ CFU mL^–1^). The bacteria were plated with a Drigalski spatula onto solidified Mueller–Hinton agar (Becton, Dickinson and Co., Sparks, MD, USA) contained in Petri dishes. The aerogel discs (*D* 1 cm) were exposed to UV light (110 V and 254 nm) for 10 min on each side, before being positioned in the inoculated Petri dishes. After 48 h, the Petri dishes were evaluated by observation of the inhibition zones (colony-free areas) and bacterial growth. All of the tests were performed in triplicate.

### Biocompatibility Assays

The cytotoxicity assay was conducted according to the ISO 0993-5:2009 protocol. Primary dermal fibroblast (HDFn) cells were cultivated at 37 °C in a humidified 5% CO_2_ atmosphere in Dulbecco’s modified Eagle’s medium (DEMEM, GIBCO) supplemented with 15% fetal bovine serum (FBS) (v/v). For the cytotoxicity assay, cells were seeded at a density of 2 × 10^4^ cells per well and stored in a heated chamber overnight before incubation with the aerogel extract. For the extract, aerogels were sterilized with UVC radiation (λ = 254 nm) for 15 min each side followed by 24 h of incubation in cell culture medium (DMEM high glucose supplemented with 15% FBS). The cell medium was replaced with the 24 h aerogel extract and the cells were incubated in the heated chamber for at least 24 h before the Resazurin assay. For the Resazurin assay, the aerogel-extract medium was replaced with DMEM high glucose containing Resazurin (20 μL, 0.6 mM) and incubated for 4 h; the absorbance was read at λ = 570 and 600 nm in a microplate reader (PerkinElmer-VICTORNivo). Cell passages were between 30 and 32. All experiments were repeated in triplicate.

### Statistical Analysis

The data were subjected to analysis of variance test, assuming a confidence level of 95% (*P* < 0.05). Statistical analyses were performed using the software R version 3.6.0.

## Results and Discussion

### Niobium Oxide and Silver Niobate Synthesis

**Figure 1:**
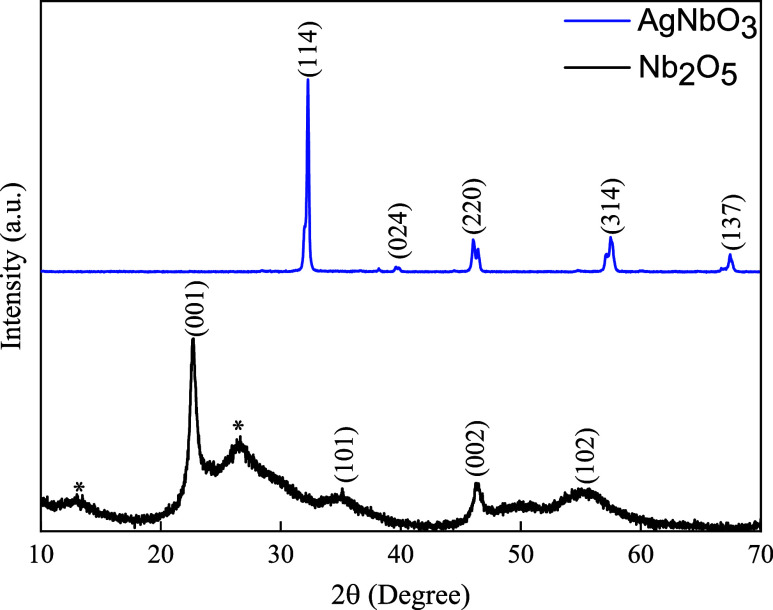
X-ray diffraction patterns with crystallographic planes of the pseudohexagonal phase of Nb_2_O_5_ and cubic phase of AgNbO_3_. * is related to Nb_2_O_5_·*n*H_2_O.

The Nb_2_O_5_ and silver niobate phase and structural formation were evaluated by X-ray diffraction (XRD) (Figure 1). Concerning the Nb_2_O_5_ synthesis, a pseudohexagonal phase was obtained according to the Joint Committee on Powder Diffraction Standards (JCPDS) number 28-0317. Two large diffraction peaks at 12 and 26° were also observed, related to hydrated niobium oxide (Nb_2_O_5_·*n*H_2_O).^35,36^ The synthesized niobium oxide was used as the precursor for silver niobate synthesis, which was successfully synthesized. A cubic phase of AgNbO_3_ (JCPDS no. 52-0405) was obtained. The XRD pattern exhibits only sharp and well-defined peaks related to AgNbO_3_ ((114), (024), (220), (314), (137)) and therefore, it is considered a crystalline pure sample.^37^

**Figure 2:**
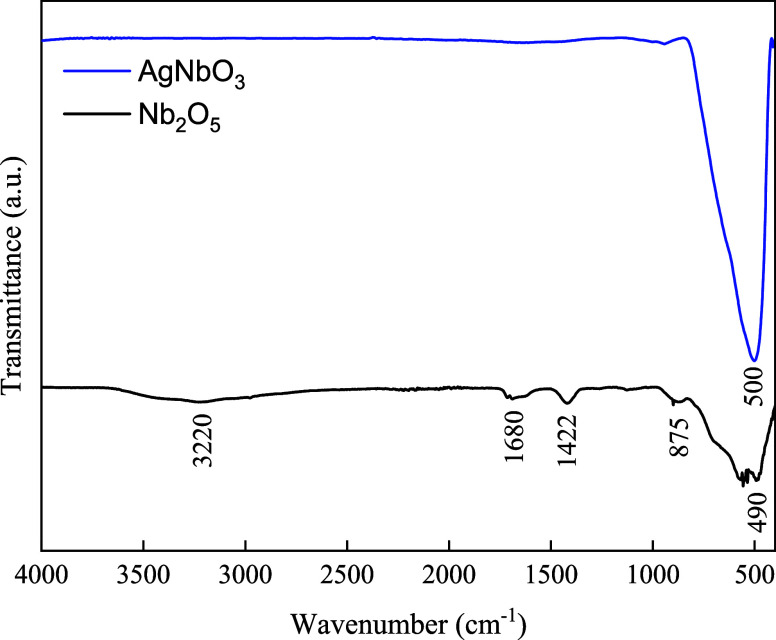
FTIR-ATR for the pseudohexagonal phase of Nb_2_O_5_ and cubic phase of AgNbO_3_.

The synthesized materials were also characterized by FTIR-ATR (Figure 2). Nb_2_O_5_ exhibits bands in the region 1000–500 cm^–1^, related to Nb–O bonding. The bands around 875–500 cm^–1^ are characteristic of the Nb=O stretch and angular vibration of Nb–O–Nb.^39^ The bands at approximately 1680 and 1422 cm^–1^ are attributed to C–O and C=O, respectively, probably related to the oxalate and carbonyl groups from the synthesis residues. The band at 3220 cm^–1^ can be attributed to the hydroxyl groups adsorbed on the surface of the sample.^35^ The presence of the residues may be responsible for the stabilization of the pseudohexagonal metastable phase of Nb_2_O_5_.^36^ Regarding the synthesis of silver niobate, the broad peak around 650–490 cm^–1^ is attributed to Nb–O stretching vibration and Nb–O–Nb bending vibration. Similarly to Nb_2_O_5_, the bands around 1000 cm^–1^ are related to Nb–O bonding. These data agree with the XRD outcome that the silver niobate was effectively synthesized using Nb_2_O_5_ as precursor.

**Figure 3:**
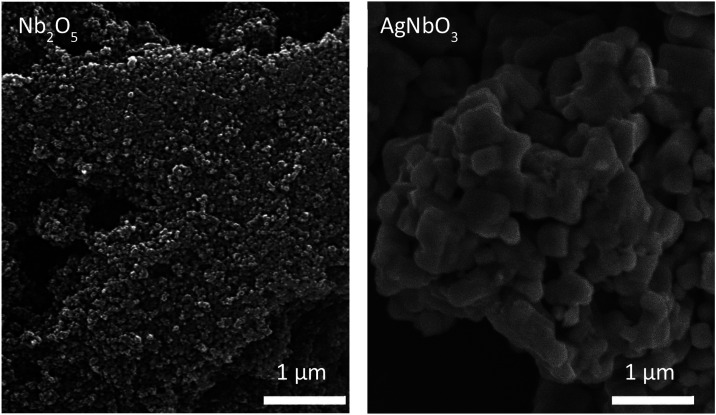
SEM micrographs of the synthesized Nb_2_O_5_ and AgNbO_3_, evidencing the different shapes and sizes of the samples.

The morphology of Nb_2_O_5_ and AgNbO_3_ was verified by SEM images (Figure 3). Nb_2_O_5_ exhibits particles with spherical morphology, in nanometric scale, with homogeneous size and morphology, indicating the efficiency of the synthesis method. Regarding AgNbO_3_ synthesis, the particles have an irregular cubic-like shape, aggregated in clusters. In both synthesized materials, no other morphologies were found, suggesting that only the desired materials were obtained. These results are coherent with those founded by Lu et al.^37^ Therefore, considering these outcomes, the synthesis of silver niobate was considered effective, and it was used for the preparation of composite cellulose-based aerogels.

### Cellulose/Silver Niobate Composite Aerogel Synthesis

**Figure 4:**
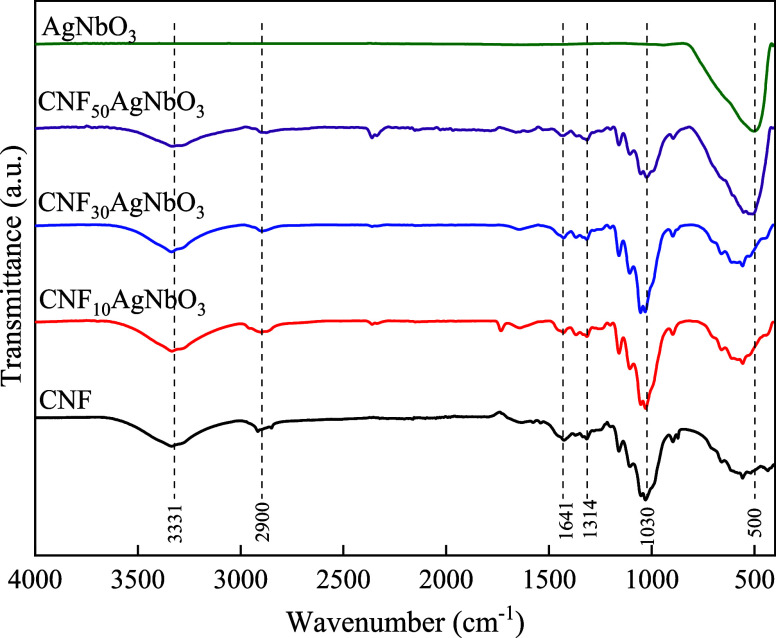
FTIR-ATR for the aerogels prepared by freeze-drying for cellulose aerogel; CNF_10_AgNbO_3_ aerogel; CNF_30_AgNbO_3_ aerogel; CNF_50_AgNbO_3_ aerogel; and AgNbO_3_. The presence of neat material bands for the composite materials can be seen.

Cellulose nanofibers were combined with the synthesized silver niobate at different concentrations (10, 30, and 50% w/w) and aerogels were obtained by freeze-drying. To verify the functional groups present at the composites, FTIR analysis was performed (Figure 4). AgNbO_3_ is characterized by the presence of a broad band around 650–490 cm^–1^, related to Nb–O stretching vibration and Nb–O–Nb bending vibration. Regarding the functional groups of cellulose, at around 3500 cm^–1^ is observed a hydrogen-bonded −OH stretching; the C–H stretching is located at 2900 cm^–1^. Approximately at 1641 cm^–1^ the band is attributed to the −OH bending of adsorbed water, while the bands around 1432 and 1314 cm^–1^ are related to CH_2_ bending and C–H bending, respectively. The bands at 1036 and 896 cm^–1^ are associated with C–H bending and CH_2_ stretching, respectively.^40^ All compositions of composite aerogels exhibit bands characteristics of CNF and the band characteristic of silver niobate shows increased intensity with increase in the ceramics content in the aerogel. Therefore, the FTIR results indicate the presence of silver niobate in cellulose aerogels, suggesting the efficient obtention of composite aerogels.

**Figure 5:**
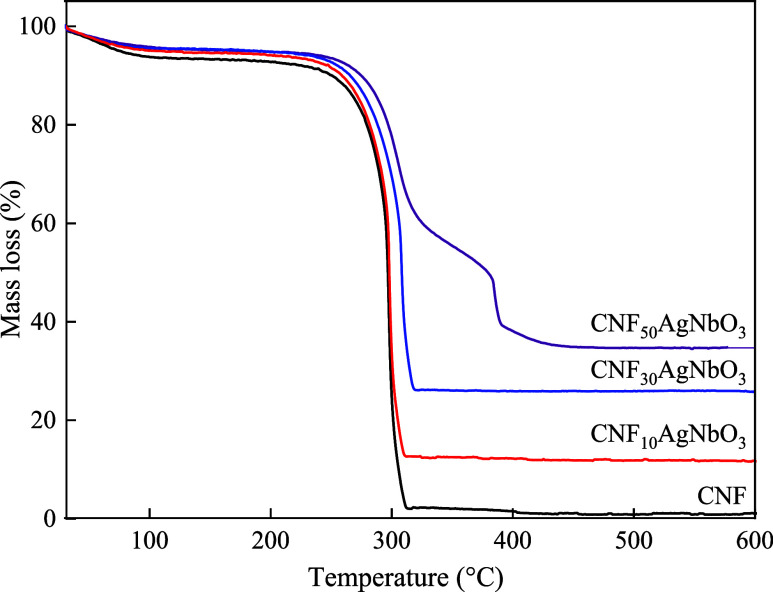
Thermogravimetric (TG) curves of cellulose aerogel; CNF_10_AgNbO_3_; CNF_30_AgNbO_3_; and CNF_50_AgNbO_3_, showing the increase at the maximum degradation temperature.

Thermogravimetric analyses were performed to verify the inorganic content of the composites and to analyze the thermal stability of the developed aerogels (Figure 5). The first weight loss for CNF occurs around 100–120 °C and is related to water evaporation.^41^ The temperature of maximum mass loss for cellulose is 305 °C and is correlated to the depolymerization, dehydration, and decomposition reactions of glycosidic units.^42^ The addition of silver niobate to the cellulose matrix increases the temperature of maximum mass loss, according to the increase of ceramics content. This may be related to the carrier effect of silver niobate on the polymer decomposition ablation products.^43^ Similar outcomes were found by Esposito et al.^44^ Regarding the concentration of silver niobate in the final cellulose-based aerogel, the nominal content of CNF_10_AgNbO_3_ and CNF_30_AgNbO_3_ (10 and 30%, respectively) corresponds to the real content. However, when 50% of AgNbO_3_ was added to the cellulose matrix, only 35% was quantified. This may be because high ceramics content may form aggregates within the polymer matrix, leading to a certain heterogeneity degree of the aerogels.

**Figure 6:**
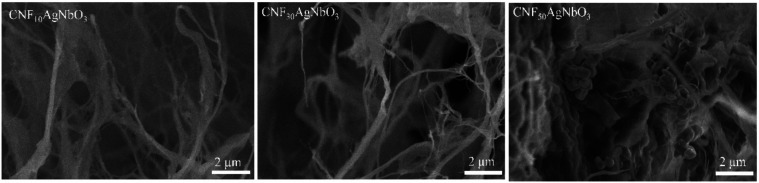
SEM images for CNF_10_AgNbO_3_; CNF_30_AgNbO_3_; and CNF_50_AgNbO_3_.

SEM analysis was performed to verify the morphology of the developed aerogels (Figure 6). The composite aerogels present a fibrillar shape due to the presence of CNF. With the increase of silver niobate concentration at the composites, it is possible to verify a second cubic phase, related to the presence of AgNbO_3_. Silver niobate is randomly distributed all over the aerogel structure. A great porosity at the aerogels is also noticed, a fundamental characteristic for application in tissue regeneration, since the porous structure of scaffolds is required for the transit of nutrients and wastes, providing a suitable environment for cell attachment, proliferation, and in-growth.^6^ For low-magnification images of these samples, see Figure S1, Supporting Information.

Contact angle measurements were performed to evaluate the influence of silver niobate content on CNF aerogel wettability, which is the ability of water to provide contact with a solid surface (Table 1, for images: Figure S2, Supporting Information). Both neat cellulose and silver niobate have a hydrophilic character. However, when the materials are combined the contact angle decreases, indicating an increase of the hydrophilicity character of the composite aerogels. This may be occurring due to the increase of the distance between the cellulose fibers caused by the presence of silver niobate, which increases the porosity of the material and allows water contact. This feature is essential for tissue replacement applications: high surface wettability is associated with increased number of adsorbed proteins on the scaffold surface, leading to superior cell adhesion and improved scaffold/cell interaction.^1^ Therefore, the developed composite aerogels have potential to be applied for tissue regeneration.

**Table 1:** Contact Angle for Composite Cellulose-Based Aerogels^a^

sample	contact angle (deg)^b^
CNF	61.7 (5.3)
CNF_10_AgNbO_3_	27.4 (2.5)
CNF_30_AgNbO_3_	26.8 (0.6)
CNF_50_AgNbO_3_	26.3 (2.8)
AgNbO_3_	78.6 (5.9)

a The numbers in parentheses are standard deviations.

b At 0.4 s using water.

### Cellulose/Silver Niobate Composite Aerogel Properties

One of the main properties for tissue regeneration is the capacity to keep a good hydration, leading to a moist environment, which will facilitate healing and the re-epithelialization process.^45^ Therefore, the swelling capacity of the aerogel, as well as water uptake and porosity, was evaluated (Table 2). Overall, the addition of silver niobate decreases the water uptake and swelling capacity, despite the increase of porosity values. The addition of 10 and 30% (w/w) of silver niobate probably increased the gap between the cellulose fibers, increasing the porosity.^46^ However, in samples with 50% (w/w) of silver niobate, the particles may decrease the porosity due to their accumulation in the space between the cellulose fibers.^47^ After 24 h of immersion on water, the composite samples showed a slight reduction of the water uptake. Similarly, the swelling behavior was reduced when silver niobate was introduced on the cellulose matrix. The swelling behavior is influenced by different factors, including the network size, intermolecular spacing, and hydrophilicity/hydrophobicity balance of the material.^48^ In the case of CNF_X_AgNbO_3_ aerogels, the silver niobate particles probably act as a cross-linking agent, reducing the flexibility of cellulose chains and the distance between the polymer chains,^49^ leading to the reduction of the swelling capacity and water uptake when compared to the neat material.

**Table 2:** Swelling Degree, Water Uptake, and Porosity for CNF and CNF_*x*_AgNbO_3_ aerogels^a^

sample	water uptake (%)	swelling (%)	porosity (%)
CNF	92.08 (1.5)	1005.98 (59.1)	16.09 (0.7)
CNF_10_AgNbO_3_	78.80 (2.2)	377.15 (50.8)	44.30 (0.5)
CNF_30_AgNbO_3_	92.11 (0.7)	1264.50 (6.8)	42.07 (2.0)
CNF_50_AgNbO_3_	64.00 (0.5)	177.89 (4.0)	5.93 (1.6)

a Numbers in parentheses are standard deviations.

### Bioactivity Performance Analysis

**Figure 7:**
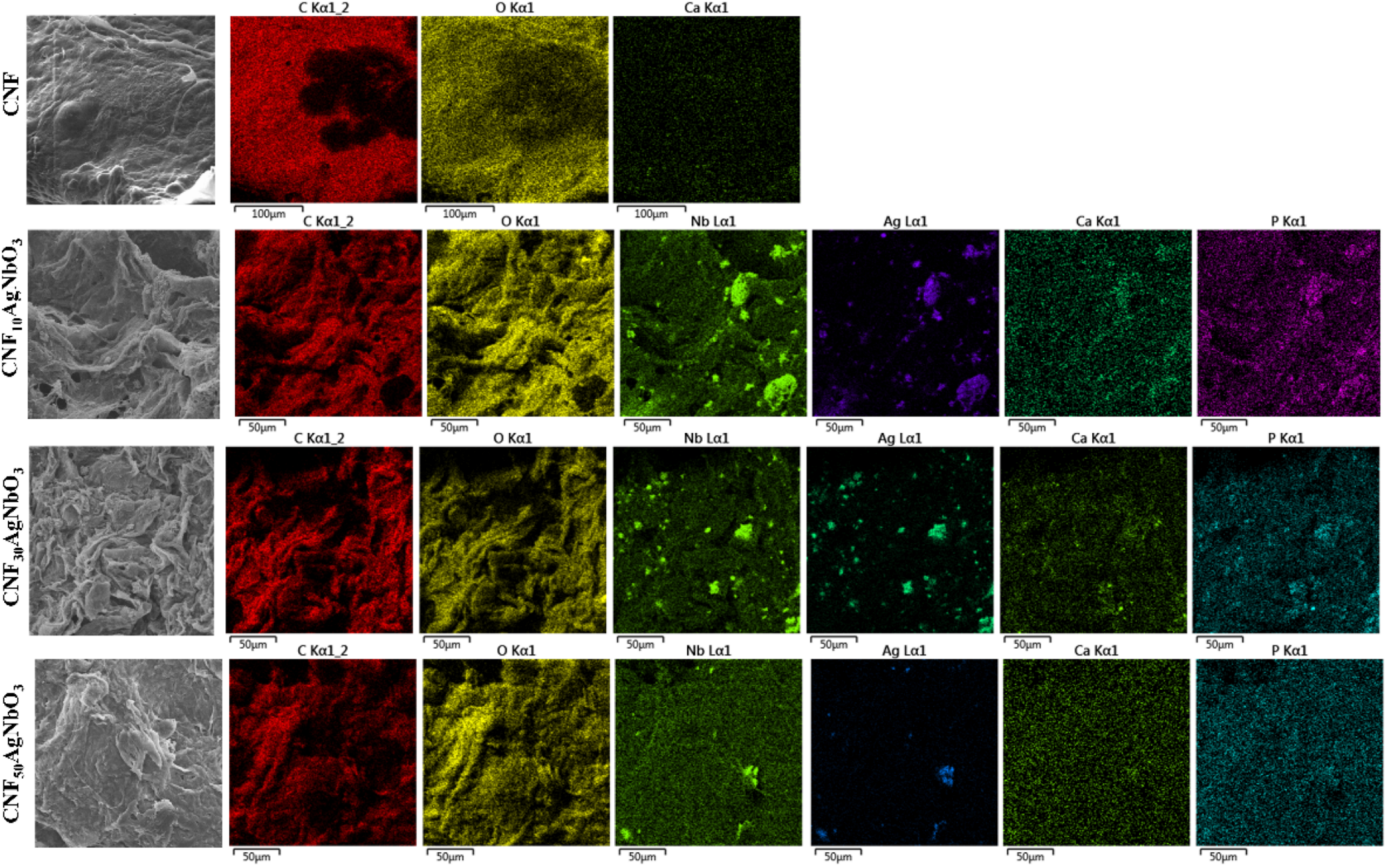
EDX elemental maps for CNF and CNF_*x*_AgNbO_3_ aerogels after immersion in SBF for 4 weeks.

The biomaterials’ activity can be monitored in vitro by the deposition of hydroxyapatite (Ca_5_(PO_4_)_3_OH) on the surface of the materials in a simulated body fluid (SBF), whose ion concentrations are nearly equal to those of human blood plasma.^50^ The developed aerogels were incubated for 4 weeks with SBF and, after this time, were examined for the deposition of hydroxyapatite, by energy-dispersive X-ray (EDX) spectroscopy (Figure 7). The aerogel with pure CNF showed just the presence of calcium, which does not indicate the deposition of hydroxyapatite. On the other hand, the composite aerogels (CNF_*x*_AgNbO_3_) showed the presence of calcium and phosphate, suggesting the formation of hydroxyapatite. These outcomes indicate the bioactivity of the composite materials, mainly due to the presence of silver niobate in the aerogel. These results agree with those found by Miyazaki et al.,^51^ that niobium compounds are bioactive. In addition, these results suggest that CNF_*x*_AgNbO_3_ are adequate materials for application in tissue engineering.

### Antibacterial Activity

**Figure 8:**
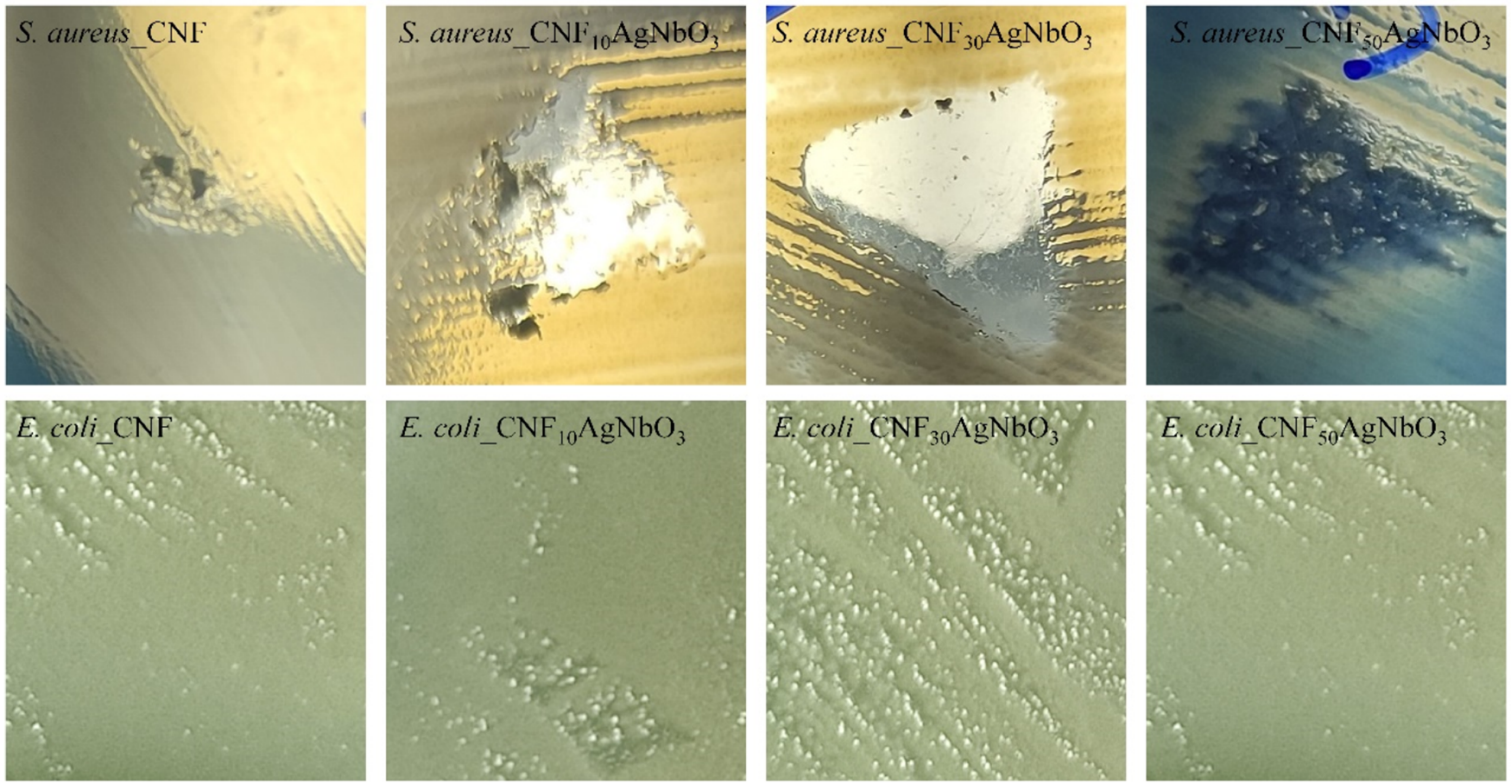
Digital photography of the disc diffusion test of the developed composite aerogels against Gram-positive bacteria (*S. aureus*) and Gram-negative bacteria (*E. coli*). It is possible to observe antibacterial activity only against *S. aureus*.

The CNF and CNF_*x*_AgNbO_3_ aerogels were examined in terms of their antimicrobial activity. All of the samples were effective in inhibiting *S. aureus* growth by contact. In other words, the places that were in contact with the developed aerogels showed no *S. aureus* growth (Figure 8). This kind of inhibition demonstrates that the antimicrobial agent is not able to be diffused on the agar medium after 24 h of incubation. Two mechanisms are responsible for the bacterial growth inhibition. In the case of pure cellulose, it can inactivate bacteria by physically damaging their cell membrane^52^ and in the case of CNF_*x*_AgNbO_3_ aerogels, samples with silver component are known to have the capacity to inhibit bacterial growth, due to the ion arrangement that can damage both the cell envelope and the bacterial genetic material. Moreover, the silver ions can interact with nucleic acids and directly with the DNA bases, leading to bacterial death.^53^ In summary, CNF_*x*_AgNbO_3_ aerogels have the capacity to inhibit bacterial growth, which is a desired characteristic for tissue regeneration application.

Regarding the effect of CNF_*x*_AgNbO_3_ aerogels on *E. coli* growth, it was observed that the materials did not affect the growth of these Gram-negative bacteria. This may be related to the bacterial resistance to silver. *E. coli* produces flagellin, a protein that can cause the aggregation of silver particles and Ag^+^ reduction, inhibiting silver contact with and entry into cells, and efflux of silver particles and Ag^+^ in cells. As a consequence, the antibacterial effectiveness of silver niobate is diminished.^54^ According to Panáček,^55^ this effect of silver aggregation is observed even if the silver particles are further stabilized using surfactants or polymers.

### In Vitro Study of Cell Viability

**Figure 9:**
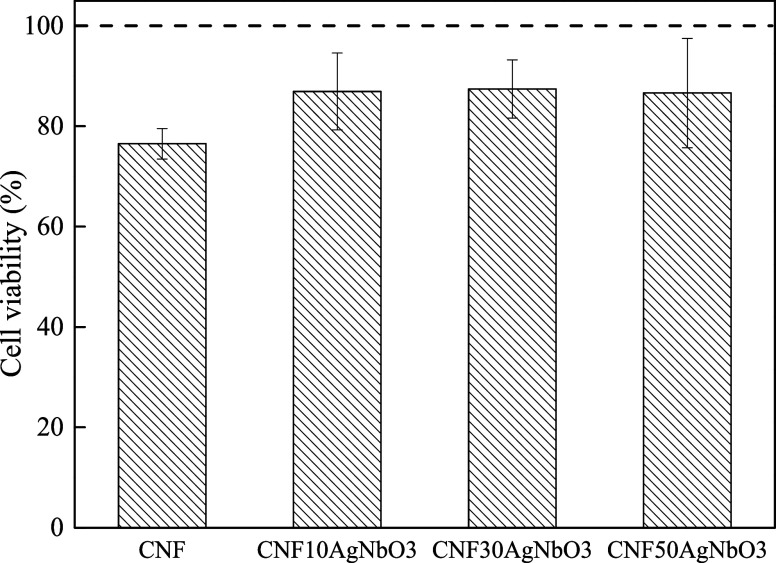
Results of the indirect cytotoxicity of CNF and composite aerogels on HDFn cells after 24 h.

Resazurin assays were performed to study the cytotoxicity of composite aerogel scaffolds. The presence of silver, even as silver niobate, could be a concern due to the potential toxicity of this element.^56^Figure 9 reveals that the composite scaffolds presented excellent biocompatibility after 24 h. The aerogels of pure CNF caused a reduction of cell viability in comparison to the composite aerogels. However, a material is considered not biocompatible when a reduction of 30% of cell viability is observed when compared to positive control.^57^ Therefore, all of the developed aerogels are biocompatible with human fibroblast cells. Similar outcomes were observed in other studies, including the absence of silver toxicity when used in composite form.^58,59^ The great biocompatibility of the developed composite aerogels indicates the potential application of these materials for tissue engineering.

## Conclusions

In this study, we developed bioactive 3D aerogels composed of cellulose nanofibers and silver niobate, with potential applications in tissue regeneration. Silver niobate nanoparticles were successfully synthesized and exhibited an irregular cubic-like morphology. The cellulose nanofiber (CNF) and CNF_*x*_AgNbO_3_ aerogels were efficiently produced through freeze-drying, resulting in a porous structure. The incorporation of silver niobate enhanced the thermal stability, hydrophilicity, and porosity of the materials—key characteristics for tissue regeneration applications. Furthermore, all of the developed aerogels demonstrated satisfactory water uptake and swelling properties. Notably, these materials inhibited the growth of *S. aureus* through direct contact. However, the same effect was not observed against *E. coli*, due to a bacterial protein production that overcomes the antibacterial effect of silver niobate. Most importantly, only the composite aerogels showed hydroxyapatite deposition on their surfaces, indicating significant bioactivity. Cytocompatibility studies revealed that the CNF_*x*_AgNbO_3_ aerogels exhibit excellent biocompatibility with human fibroblast cells. Among the various concentrations of silver niobate tested, a 30% w/w ratio relative to the mass of cellulose nanofibers displayed optimized properties for tissue regeneration applications. Future research should focus on further elucidating the interaction between cells and the developed composite aerogels, as well as exploring how these materials can promote tissue regeneration, thereby enhancing their suitability for in vivo applications.

## Supporting Information Available

Low-magnification images for cellulose and silver niobate composite aerogels, images for contact angle of the prepared composite aerogels (PDF)
