# Supplementary material for: Fabrication of Antimicrobial Cellulose and Silver Niobate Aerogels for Enhanced Tissue Regeneration
Source: ACS Omega. 2025 Apr 11;10(15):15493–502. doi: 10.1021/acsomega.5c00351 (PMC12019755; doi:10.1021/acsomega.5c00351)
Supplement: Supplementary file 1 — ao5c00351_si_001.pdf [file ao5c00351_si_001.pdf]

Supporting information for : **Fabrication of Antimicrobial Cellulose and Silver Niobate Aerogels for Enhanced Tissue Regeneration**

Marcela P. Bernardo[1]*; Mauricio Foschini[2]; Ana Carolina Costa Santos[3]; Carlos Ueira Vieira[3]; Natieli Saito[4]; Maria Eduarda Costa Mundim[4]; Osmando F. Lopes[1]; Daniel Pasquini[1]

[1]Institute of Chemistry, Federal University of Uberlândia, Av João Naves de Ávila, Uberlândia, MG, Brazil CEP 38400-902

[2] Physics Institute, Federal University of Uberlandia, Av João Naves de Ávila, Uberlândia, MG, Brazil CEP 38400-902

[3] Genetics Laboratory, Institute of Biotechnology, Federal University of Uberlandia, Rua Ceará, Uberlândia, MG, Brazil. CEP: 38402-018

[4] Biotechnology Institute, Laboratório de Nanobiotecnologia Prof. Dr. Luiz Ricardo Goulart Filho, Federal University of Uberlandia, Rua Ceará, Uberlândia, MG, Brazil. CEP: 38402-018

*Corresponding-author: marcelapiassib@gmail.com

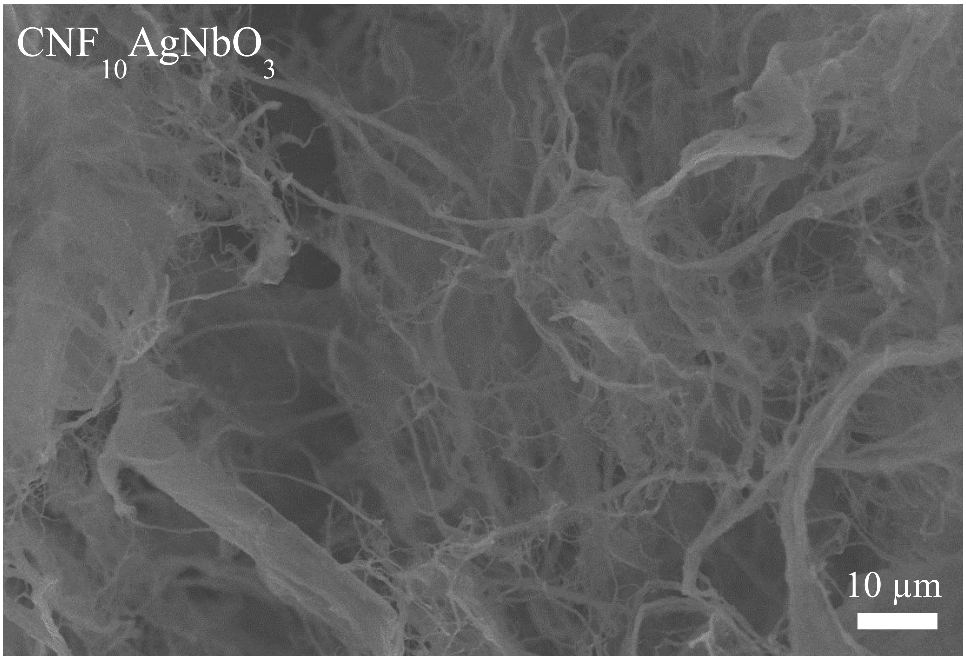

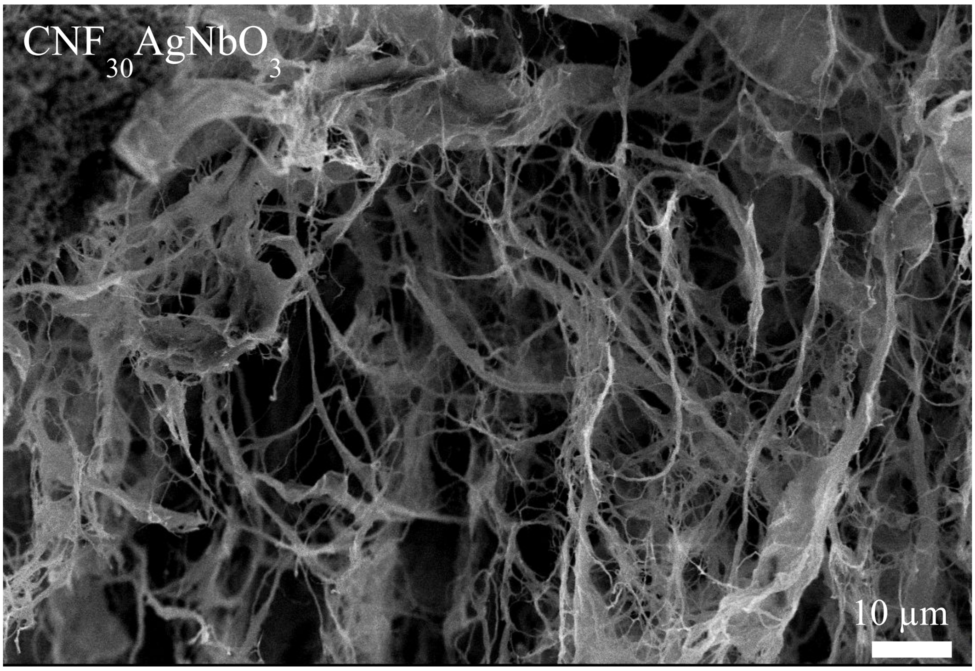

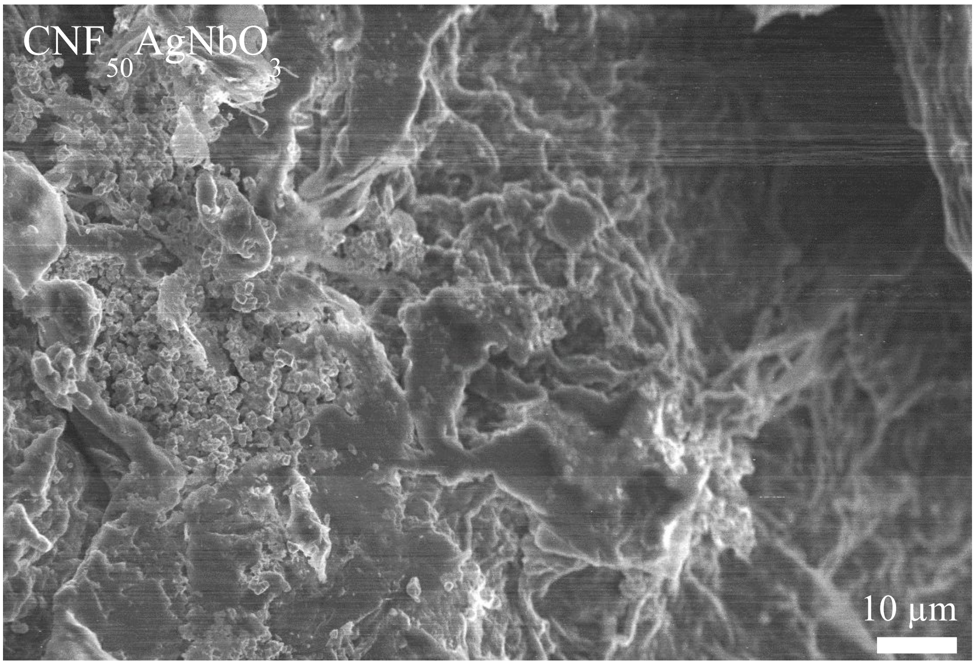

Figure S1. Low magnification images for cellulose and silver niobate composite aerogels.

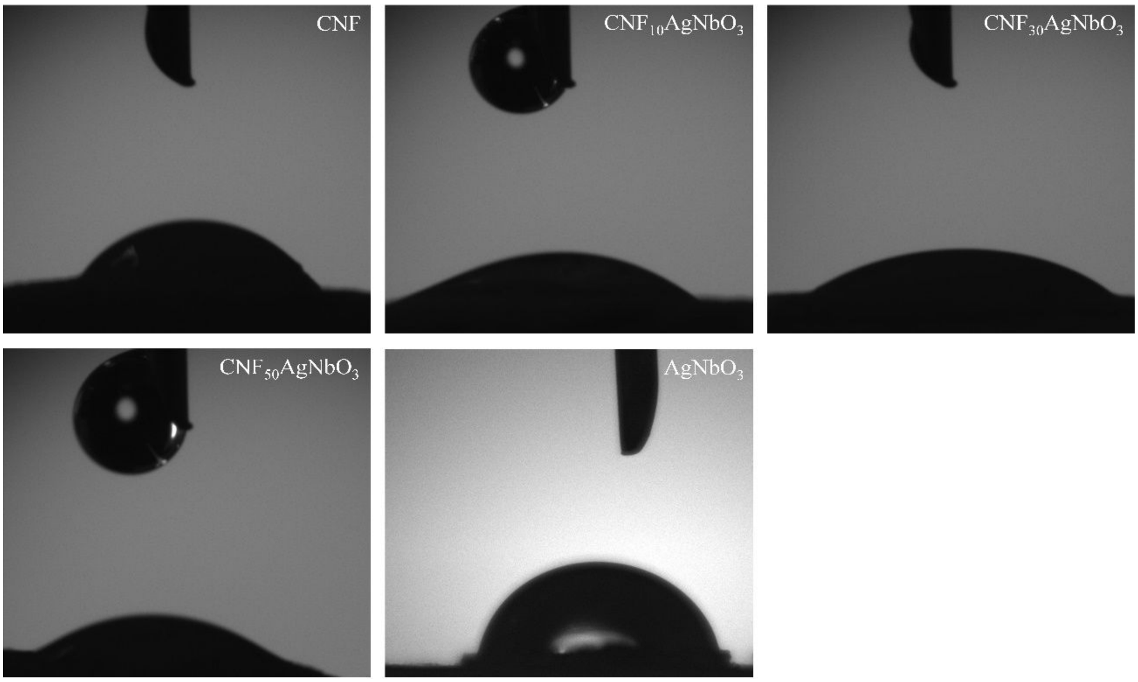

Figure S2. Contact angle images for the prepared composite aerogels.
